# Changing the Taxonomic Classification of Strain NRRL5109 to Aspergillus fumigatus

**DOI:** 10.1128/MRA.00475-21

**Published:** 2021-12-02

**Authors:** Licui Li, Zhihe Yu

**Affiliations:** a College of Life Sciences, Yangtze University, Jingzhou, Hubei, China; Vanderbilt University

## REPLY

In his Letter to the Editor (1), Dr. Houbraken suggested that Aspergillus fumigatus should still be used as the taxonomic classification of strain NRRL50109, which was classified as Aspergillus neoellipticus (2).

Strain NRRL5109 was classified as A. fumigatus var. *ellipticus* because of its elliptical conidial heads and smooth or nearly smooth walls (3). Kozakiewicz (4) first proposed changing A. fumigatus var. *ellipticus* into the species *A. neoellipticus*. Samson (5) relegated them to synonyms of A. fumigatus. Peterson (6) asserted that A. fumigatus var. *ellipticus* was supported as a species according to a phylogenetic analysis using four loci but still upheld its synonymous status.

At present, there is no definite evidence to support A. fumigatus as a separate species. Thus, we agree with Dr. Houbraken’s suggestion that the taxonomic classification of strain NRRL5109 be modified to Aspergillus fumigatus.

## References

[1] Houbraken J, Visagie CM, Frisvad JC. 2021. Recommendations to prevent taxonomic misidentification of genome-sequenced fungal strains. Microbiol Resour Announc 10:e01074-20. doi:10.1128/MRA.01074-20. PMC8638587 34854710

[2] Li LC, Hsiang T, Li QL, Wang L, Yu ZH. 2018. Draft genome sequence of NRRL 5109, an ex-type isolate of *Aspergillus neoellipticus*. Microbiol Resour Announc 7:e01262-18. doi:10.1128/MRA.01262-18. 30533860 PMC6284092

[3] Raper KB, Fennell DI. 1965. The genus Aspergillus. Williams & Wilkins, Baltimore, MD.

[4] Kozakiewicz Z. 1989. *Aspergillus* species on stored products. Mycol Papers 161:1–188.

[5] Samson RA, Hong S, Peterson SW, Frisvad JC, Varga J. 2007. Polyphasic taxonomy of *Aspergillus* section Fumigati and its teleomorph *Neosartorya*. Stud Mycol 59:147–203. doi:10.3114/sim.2007.59.14. 18490953 PMC2275200

[6] Peterson SW. 2008. Phylogenetic analysis of *Aspergillus* species using DNA sequences from four loci. Mycologia 100:205–226. doi:10.3852/mycologia.100.2.205. 18595197

